# Experimental approaches for micromagnetic coercivity analysis of advanced permanent magnet materials

**DOI:** 10.1080/14686996.2021.1874836

**Published:** 2021-02-12

**Authors:** Satoshi Okamoto

**Affiliations:** a Institute of Multidisciplinary Research for Advanced Materials (IMRAM), Tohoku University, Sendai, Japan; b Center for Spintronics Research Network (CSRN), Tohoku University, Sendai, Japan; c Elements Strategy Initiative Center for Magnetic Materials (ESICMM), National Institute for Materials Science (NIMS), Tsukuba, Japan

**Keywords:** Coercivity, magnetization reversal, thermal activation, Nd-Fe-B, 203 Magnetics, Spintronics, Superconductors

## Abstract

Although coercivity is one of the fundamental properties of permanent magnets, it has not been well understood. In this paper, micromagnetics and thermal activation magnetization reversal theories are briefly reviewed, and then our recent macroscopic and microscopic experimental approaches for thermally activated magnetization reversal in advanced Nd-Fe-B hot-deformed magnets are explained. Our experimental results are well supported by the recent atomistic spin model calculations. Moreover, the systematic micromagnetics simulation study makes much clearer the physical picture of the thermally activated magnetization reversal process in permanent magnets.

## Graphical abstract

**Figure:**
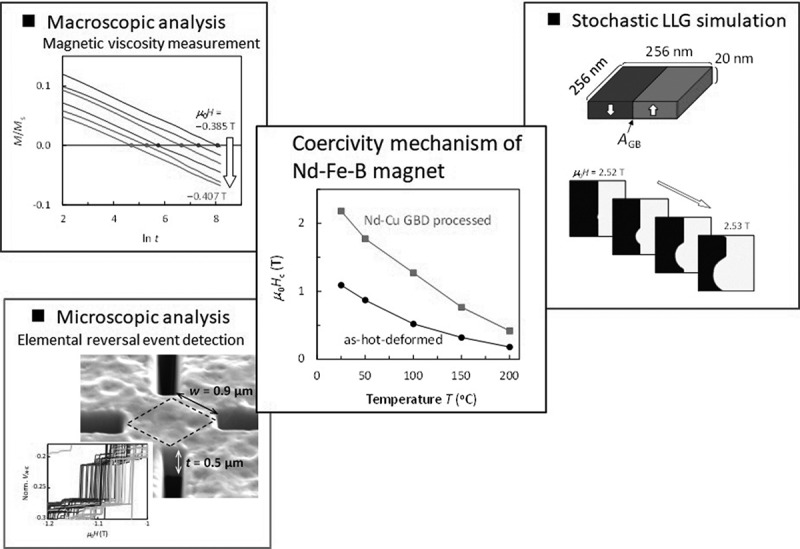

## 1 Introduction

**Figure 1.**
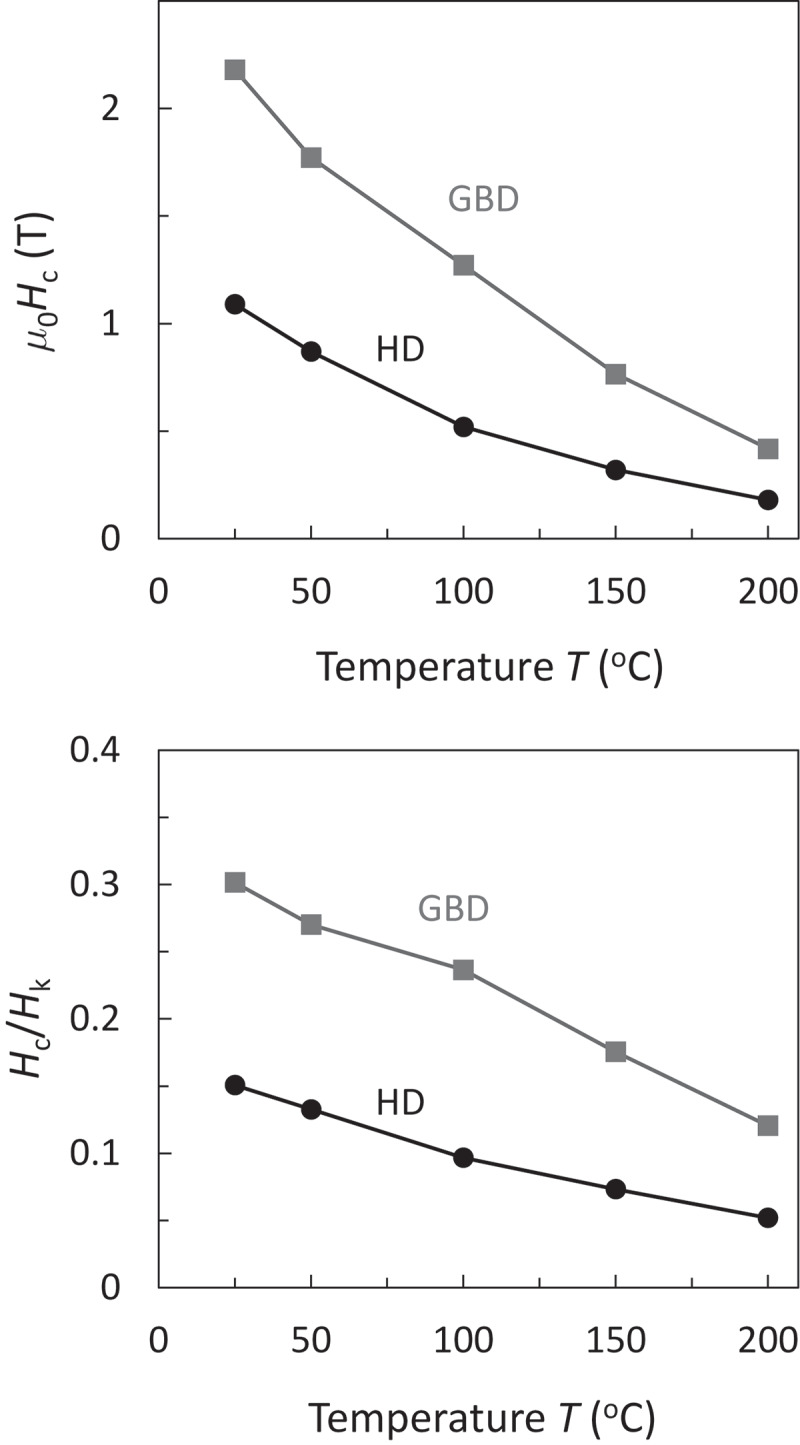
Temperature dependent (a) *m*_0_*H*_c_ and (b) *H*_c_*/H*_k_ of HD and GBD magnets [10]. These two magnets have the same size of 3 × 0.5 × 0.5 mm^3^ with c-axis parallel to the long axis

The development of high-performance Nd-Fe-B magnets without using heavy-rare-earth (HRE) elements has been a critical issue for the traction motor application of electric/hybrid vehicles [1,2]. During the past decade, various kinds of HRE-free Nd-Fe-B magnets have been developed through elaborate microstructure control processes, such as hot-deformation [3], eutectic alloy grain-boundary diffusion [4,5], He-jet-milling press-less processes [6,7], Ga-added and optimal heat-treatment process [8,9], and so on. As an example, Figure 1(a) shows the coercivity *H*_c_ of two types of hot-deformed magnets, which are the as-hot-deformed (HD) and Nd-Cu eutectic alloy grain-boundary diffused (GBD) ones, as a function of temperature [10]. Figure 1(b) shows their *H*_c_/*H*_k_ as a function of temperature, where *H*_k_ is the anisotropy field, which has been regarded as the ideal upper limit of coercivity. Although the GBD magnet exhibits an approximately two-times larger *H*_c_ than that of HD magnet, it remains at one third of *H*_k_. These small values of *H*_c_/*H*_k_ have been commonly observed in various permanent magnets, as pointed out by Kronmüller [11]. Moreover, the *H*_c_ values of both magnets significantly decrease with increasing temperature. The values of *H*_c_*/H*_k_ also decrease with increasing temperature. These facts indicate that the reduction of *H*_c_ with temperature cannot be explained only by the reduction of the magnetic anisotropy constant. Therefore, the magnetization reversal process and its change with temperature are essentially important.

The study on the magnetization reversal process of permanent magnets has a long history. About three-quarters of a century ago, Brown pointed out the theoretical difficulties on this issue [12]. Thus, the lower value of *H*_c_ compared with *H*_k_ is now referred to as the Brown’s paradox. When we discuss the coercivity, we usually measure the field at which the magnetization becomes zero on the magnetization curve at ambient conditions. However, the measured coercivity is the consequence of many elemental processes including nucleation and domain wall depinning/propagation. Both nucleation and domain wall depinning are the breaking events of the equilibrium states. These equilibrium breaking events are initiated by the formation of a small reversed embryo with a size of the order of the exchange length. In this size range, the thermal activation process plays a significant role even in the bulk magnet. In fact, these pictures of magnetization reversal in permanent magnets have been intensively studied by Givord et al. [13–18]. However, there are some ambiguous theoretical points. The most important issue that has not been discussed before is the relationship between the experimentally analyzed *H*_c_, which is macroscopically measured, and the actual microscopic reversal events.

Very recently, theoretical approaches for the microscopic reversal events have advanced significantly. The energy barrier for the thermally activated nucleation was computationally evaluated from the energy landscape calculation with the energy minimizing path method [19,20]. More accurately, the thermally activated nucleation process and its energy barrier were successfully calculated using the atomistic spin model [21–23]. From the experimental approaches on this issue, we re-examined the micromagnetic coercivity analysis [10,24]. Moreover, the detection of elemental magnetization reversal events and their thermal fluctuation behaviors were successfully performed [25,26].

In this paper, the recent developments of experimental approaches for the magnetization reversal process are reviewed. The micromagnetics and thermal activation theories on the magnetization reversal processes for permanent magnets are briefly reviewed in Section 2. In Section 3, the experimental approaches for the thermally activated magnetization reversal processes are explained from the macroscopic and microscopic measurements. Moreover, stochastic simulation results are explained. Section 4 is the summary.

## 2. Micromagnetics and thermal activation theories for permanent magnets

### 2.1 Micromagnetics

**Figure 2.**
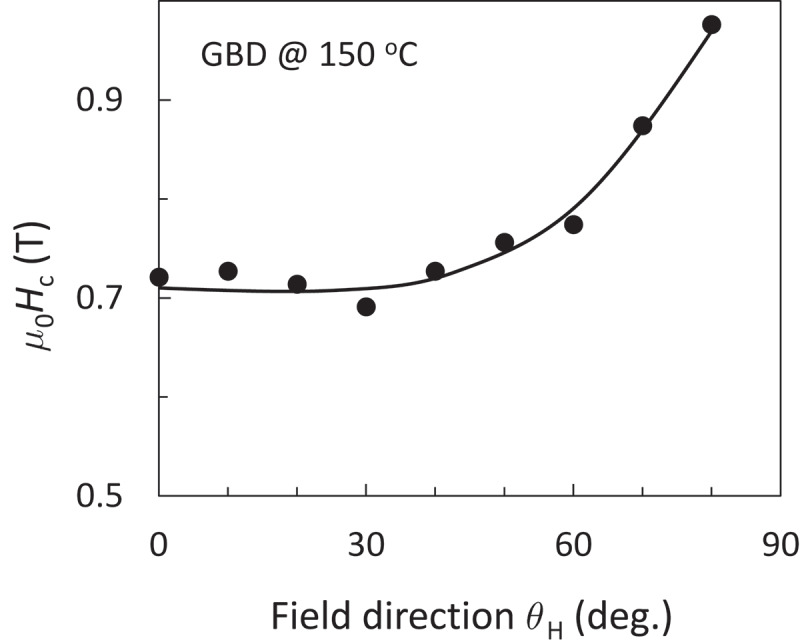
Angular dependent *m*_0_*H*_c_ of GBD magnet measured at 150°C [10]. Solid line is a guide to the eye

Micromagnetics is the mathematical energy-minimization method used to find the equilibrium magnetization state of a finite magnet body, which was originally developed by Brown [27]. Based on this approach, Aharoni formulated the curling- and buckling-type nucleation processes for spheroids and infinite cylinders with a size larger than a certain critical diameter *d*_c_ [28–30]. For a sphere, *d*_c_ is given as [31],

(1) $$d_{c}=2l_{ex}\;q/\sqrt{N_{x}}\;,$$

where $l_{ex}=\sqrt{2A/\mu_{0}{M_{s}}^{2}}$ is the exchange length, *q* is the geometrical factor approximately given as 2, *N*_x_ is the demagnetization factor of the orthogonal direction, *A* the exchange stiffness, *M*_s_ the saturation magnetization, *μ*_0_ the permeability in vacuum. When the grain size is larger than *d*_c_, the magnetization reversal process changes from coherent rotation to nucleation. The value of *d*_c_ for Nd_2_Fe_14_B is evaluated to be as 18 nm. It is widely accepted that the reduction of the grain size in Nd-Fe-B sintered magnets effectively enhances *H*_c_ [1,2]. Someone may explain that the enhancement of *H*_c_ with decreasing the grain size is attributed to the change in the magnetization reversal process from incoherent to coherent modes. However, the experimentally discussed grain size is about μm range, which is two orders of magnitude larger than the value of *d*_c_. Therefore, it is plausible that the experimentally observed *H*_c_ enhancement with decreasing the grain size results from another mechanism rather than the change in the magnetization reversal process. The nucleation field for the grain diameter *d* > *d*_c_ is given in the curling model as [31],

(2) $$H_{n}=H_{k}-N_{z}M_{s}+\frac{4{l_{ex}}^{2}}{d^{2}}q^{2}M_{s},$$

where *N*_z_ is the demagnetization factor along the external field. Here, *H*_n_ = *H*_k_ – *N*_z_*M*_s_ is the lowest nucleation field in the curling model for an extremely large *d*. Thus, the nucleation field decreases from *H*_k_, whereas the reduction is not sufficient to fill the gap between the experimentally observed *H*_c_ and *H*_k_. The curling model assumes that the magnetic material is uniform. However, nonuniform magnetic materials including defects and/or grain boundaries have been treated as a one-dimensional model, which is a planar soft magnetic layer sandwiched between two hard magnetic layers [32–36]. By using this model, domain wall depinning and defect-driven nucleation could be theoretically calculated. Through this approach, Kromüller et al. formulated the following simple equation as [11,37,38],

(3) $$H_{c}=\alpha H_{k}-N_{eff}M_{s},$$

where *α* is the reduction coefficient related to the soft-region magnetic anisotropy and/or easy axis orientation, and *N*_eff_ is the effective local demagnetization coefficient. Kronmüller et al. studied the physical mechanism for the various cases and found that *α* was proportional to *r*_0_/*δ*_B_, where *r*_0_ is the thickness of the soft magnetic phase and *δ*_B_ is the domain wall thickness of the hard magnetic phase. Eq. (3) has been widely accepted by experimental researchers to analyze the temperature-dependent *H*_c_. By plotting *H*_c_/*M*_s_ versus *H*_k_/*M*_s_, *α* and *N*_eff_ are determined by the slope and the *y*-axis intercept, respectively. This determination assumes that *α* is invariant against temperature. However, *α* ∝ *r*_0_/*δ*_B_, originally given by Kronmüller, obviously exhibits the temperature dependence. Moreover, many experimental researchers blindly accept the nucleation process when adopting Eq. (3) to the experimental *H*_c_. However, from the experimental results of angular-dependent *H*_c_, a 1/cos*θ*_H_ type behavior has been observed in various magnets, including Nd-Fe-B sintered, SmCo_5_ sintered, and ferrite magnets [14], where *θ*_H_ is the external field direction. Figure 2 shows an example result for GBD magnet [10]. The 1/cos*θ*_H_ type *H*_c_ behavior is explained well as the dominant magnetization reversal process of domain wall depinning. However, we believe that the actual magnetization reversal is the multiple and simultaneous events of nucleation and domain wall depinning. Therefore, this kind of alternative choice of nucleation and domain wall depinning is too simple to describe the actual magnetization reversal process in permanent magnets.

### 2.2 Thermal activation model

The micromagnetics theory explained above does not consider the thermal activation process. However, the thermal activation process plays an important role in the magnetization reversal process even in bulk magnets. This was classically evidenced by the magnetic viscosity experiments [39]. The magnetic viscosity is the phenomenon in which the magnetization *M* gradually changes with time *t* under a constant magnetic field and is empirically described as,

(4) $$M\left(t\right)\;=M\left(0\right)-Slnt$$

where *S* is the magnetic viscosity coefficient. *S* is represented by using the fluctuation field *H*_f_ and the irreversible magnetic susceptibility *χ*_irr_ as [40],

(5) $$S=\chi_{irr}H_{f}$$

The magnetic viscosity is an ensemble of stochastically occurring elemental magnetization reversal events. The probability *P*(*H*) of each event is expressed by the Néel-Arrhenius relaxation law as,

(6) $$P\left(H\right)\;=\;1-exp(-t/\tau \left(H\right))$$

where *τ* is the relaxation time given as,

(7) $$1/\tau \left(H\right)\;=f_{0}exp(-E_{b}\left(H\right)/k_{B}T)$$

where *f*_0_ is the attempt frequency, *k*_B_ is the Boltzmann constant, and *T* is the temperature. *E*_b_(*H*) is the energy barrier generally given as,

(8) $$E_{b}\left(H\right)\;=E_{0}(1-H/H_{0}{)}^{n}$$

where *E*_0_ and *H*_0_ are the barrier height at *H* = 0 and the intrinsic magnetization reversal field, respectively. *n* = 1 ~ 2 is the constant depending on the magnetization reversal process. The actual magnetic material has a certain amount of *E*_b_ dispersion. When the *E*_b_ dispersion is wider than the thermal energy *k*_B_*T, H*_f_ is given as [13],

(9) $$H_{f}=-\frac{k_{B}T}{\partial E_{b}/\partial H}.$$

Givord pointed out the experimental fact that *S* and *χ*_irr_ exhibit identical behaviors against *H* for various permanent magnets [14–16,39], and *H*_f_ can be treated as a constant from Eq. (5). Thus, *E*_b_ is expressed as a linear function against *H* from Eq. (9), resulting in *n* = 1 in Eq. (8). For the normal magnetization curve measurements which have a data acquisition time of several seconds, *E*_b_ corresponds to be 25*k*_B_*T* from Eq. (7). Moreover, assuming the effective reversal field *H* =* H*_c_ + *N*_eff_*M*_s_, Givord derived the following form of *H*_c_ from Eq. (8) with *n* = 1 [14],

(10) $$H_{c}=\frac{E_{0}}{M_{s}v_{act}}-N_{eff}M_{s}-25H_{f},$$

where *v*_act_ = *k*_B_*T*/*M*_s_*H*_f_ is called as activation volume [40]. Assuming *E*_0_ ∝ *γ*_w_*δ*_w_^2^ and *v*_act_ ∝ *δ*_w_^3^, the first term on the right-hand side of Eq. (10) becomes in proportion to *H*_k_, where *γ*_w_ is the domain wall energy ∝$\sqrt{AK_{u}}$ and *δ*_w_ is the domain wall width ∝$\sqrt{A/K_{u}}$. Thus, Eq. (10) is similar to Eq. (3), originally proposed by Kronmüller. Note that Eq. (10) is derived by assuming *n* = 1 in the energy barrier function of Eq. (8). Very recently, we proposed the more general analysis of thermally activated magnetization reversal based on magnetic viscosity measurements, as explained in the next section.

## 3. Experimental magnetization reversal analyses for permanent magnets

### 3.1 Magnetic viscosity analyses

As mentioned above, Givord discussed the thermally activated magnetization reversal by assuming the value of *n* = 1 in the energy barrier function of Eq. (8). However, El-Hilo et al. pointed out that the calculated *H*_f_ for the assembly of Stoner-Wohlfarth particles, which has *n* = 2 of Eq. (8), becomes almost constant against *H* when the *E*_b_ distribution is large [41]. This result indicates that we would have the wrong conclusion of *n* = 1 even though the actual value of *n* = 2. Since the value of *n* significantly affects the evaluated values of *E*_0_ and *H*_0_, the experimental determination of *n* is very important.

El-Hilo’s study also indicates that the calculated *H*_f_ at *H* ≈ *H*_c_ is insensitive to the *E*_b_ distribution. This result means that the evaluation of *H*_f_ at *H* ≈ *H*_c_ is reliable even if the *E*_b_ distribution exists. Conventionally, *H*_f_ has been evaluated from the separately measured *S* and *χ*_irr_ based on Eq. (5). In contrast, El-Hilo also proposed the *H*_f_ evaluation only from the magnetic viscosity measurements as,

(11) $$H_{f}=-\frac{\Delta H}{\Delta \ln\left(S/t\right)}.$$

**Figure 3.**
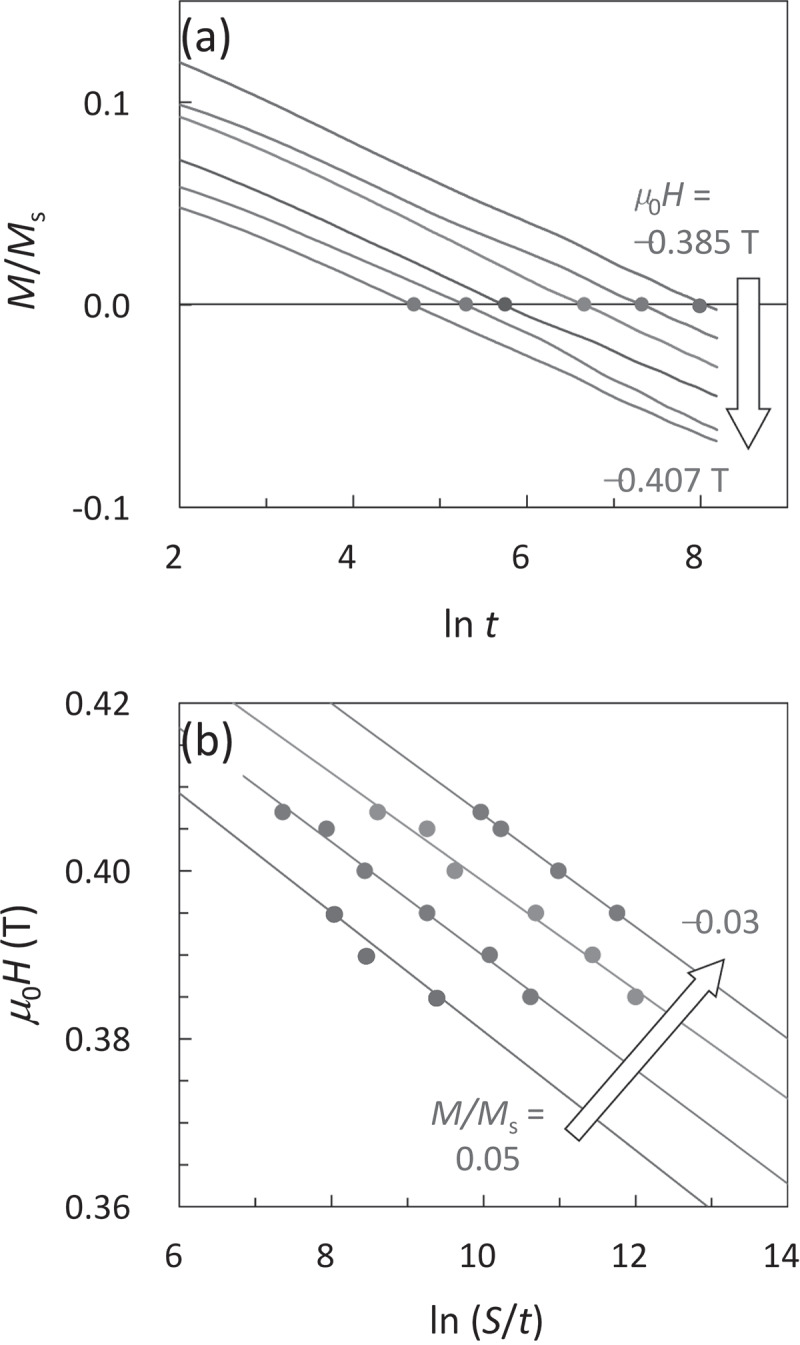
(a) Magnetic viscosity curves of GBD magnet measured at 200°C under various values of *m*_0_*H* near *m*_0_*H*c [10]. Solid marks in (a) correspond to time dependent *m*_0_*H*c. (b) H versus ln(*S/t*) for each constant *M/M*s evaluated from (a)

This evaluation of *H*_f_ is expected to have a higher accuracy than the conventional method because the measurement of *χ*_irr_ is unnecessary. As an example, the magnetic viscosity curves of the GBD magnet for various *H* around *H*_c_ are shown in Figure 3(a). From these viscosity data, *H* versus ln(*S*/*t*) are plotted in Figure 3(b). From this plot, *H*_f_ is evaluated using Eq. (11). We have experimentally confirmed that the value of *H*_f_ from Eq. (11) is identical to that from Eq. (5) [24].

**Figure 4.**
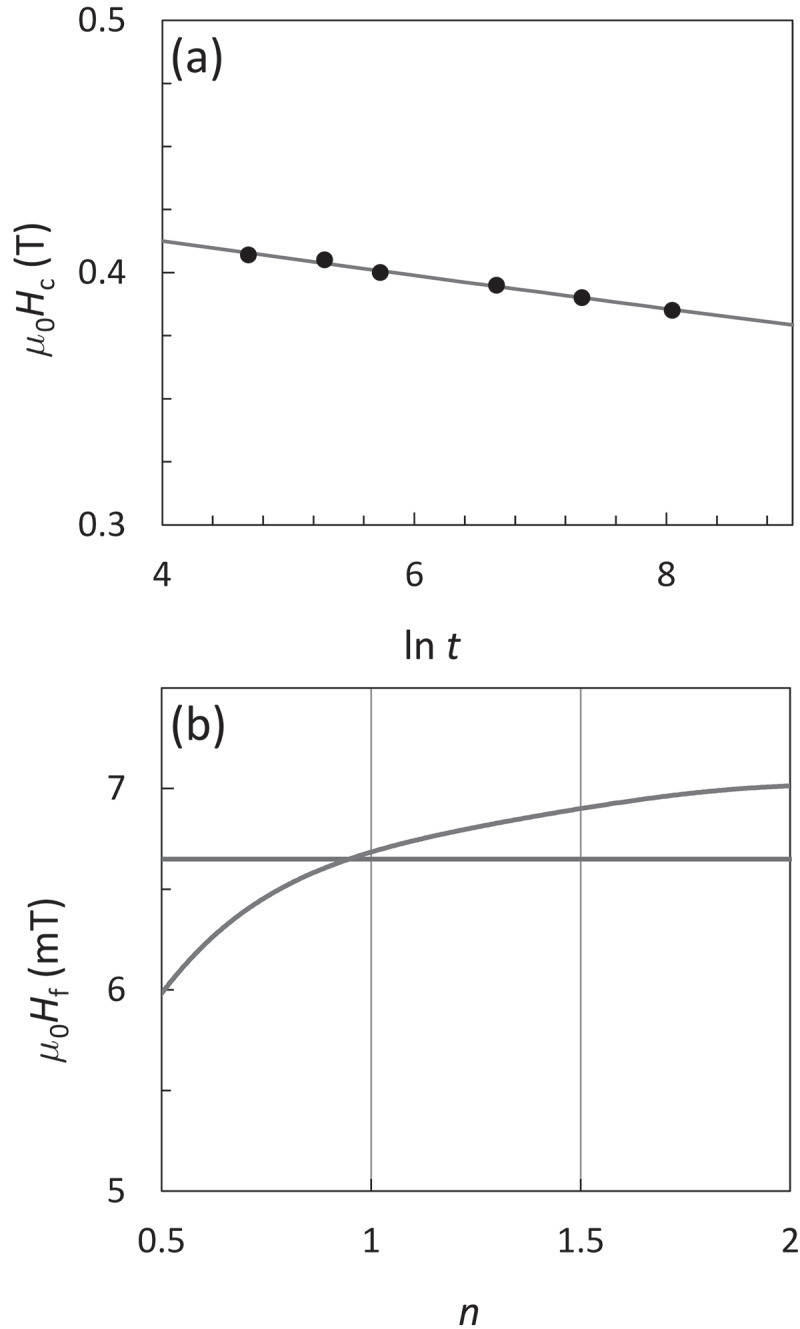
(a) Time dependent *m*_0_*H*_c_ obtained from Fig. 3(a). Solid line is the best fitting of Eq. (12). (b) *m*_0_*H*_f_ (red curve) evaluated from the time dependent *m*_0_*H*_c_ in (a) as a function of *n* of Eq. (12). Blue line is the value of *m*_0_*H*_f_ evaluated from the viscosity experiment (Fig. 3(b))

The intersection points of the viscosity curves with the transverse line of *M*/*M*_s_ = 0 in Figure 3(a) correspond to the time dependent *H*_c_, as plotted in Figure 4(a). This is formulated from *P*(*H*) = 0.5 of Eq. (6) as,

(12) $$H_{c}\left(t\right)=H_{0}\left[1-{\left\{\frac{k_{B}T}{E_{0}}\ln\left(\frac{f_{0}t}{\ln\left(2\right)}\right)\right\}}^{1/n}\right]\;.$$

This equation was first proposed by Sharrock [42,43]. The value of *f*_0_ = 1 × 10^11^ Hz is used in this study [10,44,45]. The solid line in Figure 4(a) is the best fitting result. The determination of *n* is difficult from this fitting because the fitting of Eq. (12) is possible for a certain range of *n*. Therefore, the values of *E*_0_ and *H*_0_ are evaluated by varying *n*, and then the *H*_f_ curve against *n* from Eq. (9) is obtained, as shown by the red line in Figure 4(b). The value of *H*_f_ should agree with that from the magnetic viscosity curve analysis, as shown by the blue line in Figure 4(b), consequently all the parameters of *E*_0_, *H*_0_, and *n* are fixed.

**Figure 5.**
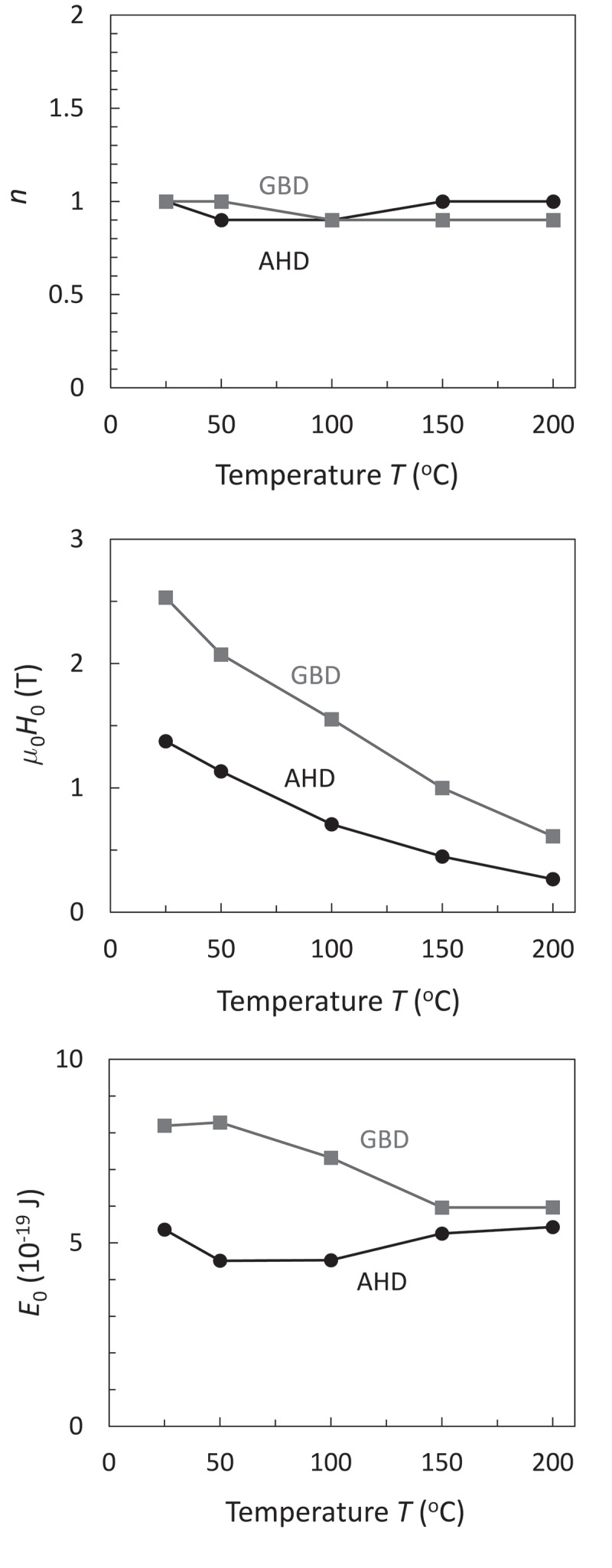
Temperature dependent (a) *n*, (b) *m*_0_*H*_0_, and (c) *E*_0_ of HD and GBD magnets [10]

Figure 5 shows the values of *E*_0_, *H*_0_, and *n* for HD and GBD magnets at various temperatures. As shown in Figure 1(a), these two magnets exhibit quite different *H*_c_, and their temperature dependences are quite large. The values of *H*_0_ exhibit the similar trends of *H*_c_ for these two magnets, whereas the values of *n* are almost 1 and insensitive to the samples and temperature. Eventually, the assumption of *n* = 1 by Givord [14] is verified. Previously, the value of *n* reflects the magnetization reversal process, i.e., *n* = 2 for coherent rotation and *n* = 1 for weak domain wall pinning [13,46]. Recently, however, *n* = 1 is supported theoretically when the two following conditions are fulfilled. One is a sufficiently large magnet body compared with the exchange length, and the second is a sufficiently slow magnetization reversal compared with the relaxation of magnetization [47]. Very recently, Toga rigorously verified this picture using the energy landscape calculation based on the atomistic spin model [23]. Obviously, these two conditions are quite reasonable for magnetization reversal in permanent magnets irrespective of the magnetization reversal processes, i.e., nucleation or domain wall depinning.

Interestingly, the values of *E*_0_ are on the order of 10^−19^ J and are almost insensitive to temperature. This value of *E*_0_ is on the same order as *γ*_w_*δ*_w_^2^, clearly indicating that the nucleation or domain wall depinning initiated from the formation of the reversed embryo with the domain wall width. More details on *E*_0_ are discussed in the next section.

### 3.2 Detection of elemental magnetization reversal events

**Figure 6.**
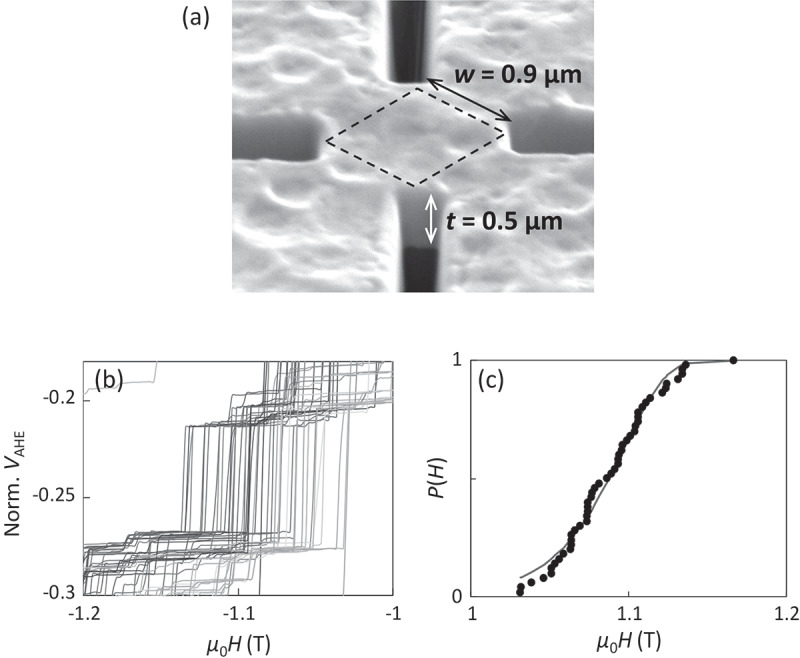
(a) AHE cross-shaped Nd-Fe-B hot-deformed magnet fabricated by FIB. (b) One step pattern of repeatedly measured 50 AHE curves. (c) Probability curve *P(H)* of the thermally fluctuated magnetization reversal of the step in (a). Blue curve in (b) is the best fitting of eq. (13) [26]

Thermally activated magnetization reversal is discussed in the preceding section. As mentioned in Section 1, however, the macroscopically measured *H*_c_ is the consequence of numerous elemental magnetization reversal events. Therefore, it is essential to detect the elemental magnetization reversal events directly. Very recently, we have directly detected elemental magnetization reversal events in Nd-Fe-B hot-deformed magnets [25,26]. With careful evaluation of the process damage on the magnetic properties, a submicron cross-shaped pattern of Nd-Fe-B hot-deformed magnet was fabricated using mechanical polishing and focused-ion beam (FIB), as shown in Figure 6(a). The *c*-axis of Nd_2_Fe_14_B is along the plane normal. The magnetic signal from this extremely small cross-center area was sensitively detected using anomalous Hall effect (AHE) measurement with a sweeping *H*. When the cross-center area is on the order of 10 μm square, the AHE curve is the same as that of the unpatterned sample. The AHE curve for the sample shown in Figure 6(a), however, becomes a staircase in which each step corresponds to the elemental magnetization reversal event. Figure 6(b) shows an example of one step of the AHE curve repeatedly measured 50 times. The step height corresponds to one or two grain magnetization reversal. Note that the reversal field of this step fluctuates about 0.1 T. Figure 6(c) shows the reversal probability *P*(*H*) as a function of *H*. This can be fitted well with the following function derived from the integration of the Néel-Arrhenius relaxation law of Eq. (6) for a constant field sweep rate *R*,

(13) $$P(H)=1-\exp\left\{-\frac{f_{0}\exp\left[-\left(E_{0}/k_{B}T\right){\left(1-H/H_{0}\right)}^{n}\right]}{n\left(R/H_{0}\right)\left(E_{0}/k_{B}T\right){\left(1-H/H_{0}\right)}^{n-1}}\right\}.$$

As mentioned above, the value of *n* = 1 is adopted for the fitting in Figure 6(c), and *f*_0_ = 1 × 10^11^ Hz is used. This very good reproducibility of *P*(*H*) curve clearly evidences that the experimentally observed step fluctuation is caused by thermal fluctuation. Thus, the values of *E*_0_ and *H*_0_ are evaluated for each step of the AHE curve.

**Figure 7.**
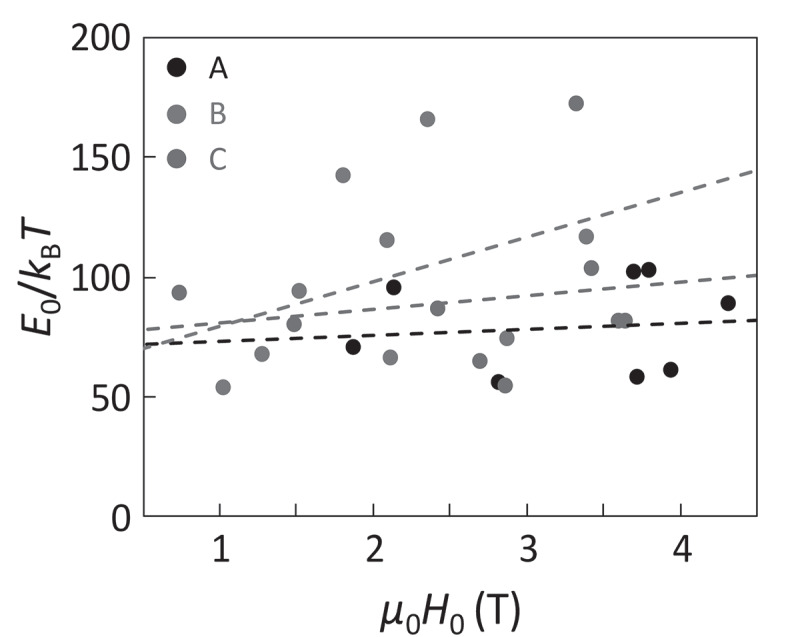
Relationship between *E*_0_/*k*_B_*T* and *H*_0_ for Samples A, B, and C. Broken lines are the linear fittings as eye guides [26]

This analysis is adopted for the three Nd-Fe-B hot-deformed magnets (Samples A ~ C) with different *μ*_0_*H*_c_ values of 2.0, 1.8, and 2.2 T. Sample A of Nd_23.4_Pr_7.5_Fe_bal._Co_3.5_B_0.9_Ga_0.5_ (wt. %) is regarded as the standard among them. Sample B of Nd_22.1_Pr_7.0_Fe_bal._Co_3.5_B_0.9_Ga_0.5_ (wt. %) has a somewhat lower Nd composition than Sample A. Sample C is the Nd-Cu eutectic alloy grain-boundary diffusion processed magnet of Sample A. For each magnet, eight or nine steps are analyzed, and the relationships between *E*_0_ and *H*_0_ are plotted in Figure 7. Although there are large dispersions of the data points, some trends can be found. First, very wide dispersion of *H*_0_ is found. Second, the slope of *E*_0_ against *H*_0_ for Samples A and C is small whereas it becomes large for Sample B, as depicted by the broken lines in Figure 7.

**Figure 8.**
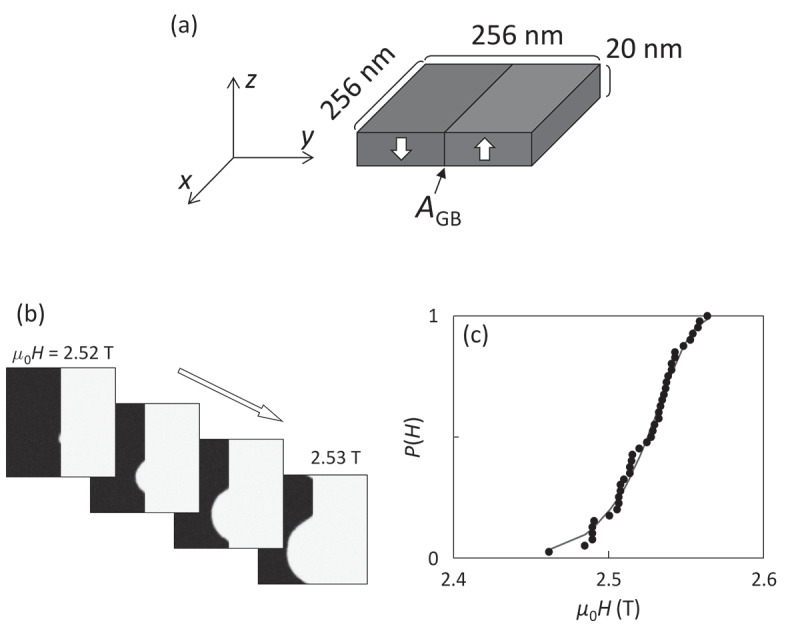
(a) Schematic illustration of the two-grain model using in the LLG simulation. *A*_GB_ is normalized intergrain exchange stiffness with respect to the exchange stiffness of Nd_2_Fe_14_B (b) Calculated snapshot images of the z-component of magnetization for the domain-wall depinning process with continuously varying field *H*. (c) Probability curve *P(H)* of the thermally fluctuated domain-wall depinning. Blue curve in (c) is the best fitting of eq. (13) [26]

**Figure 9.**
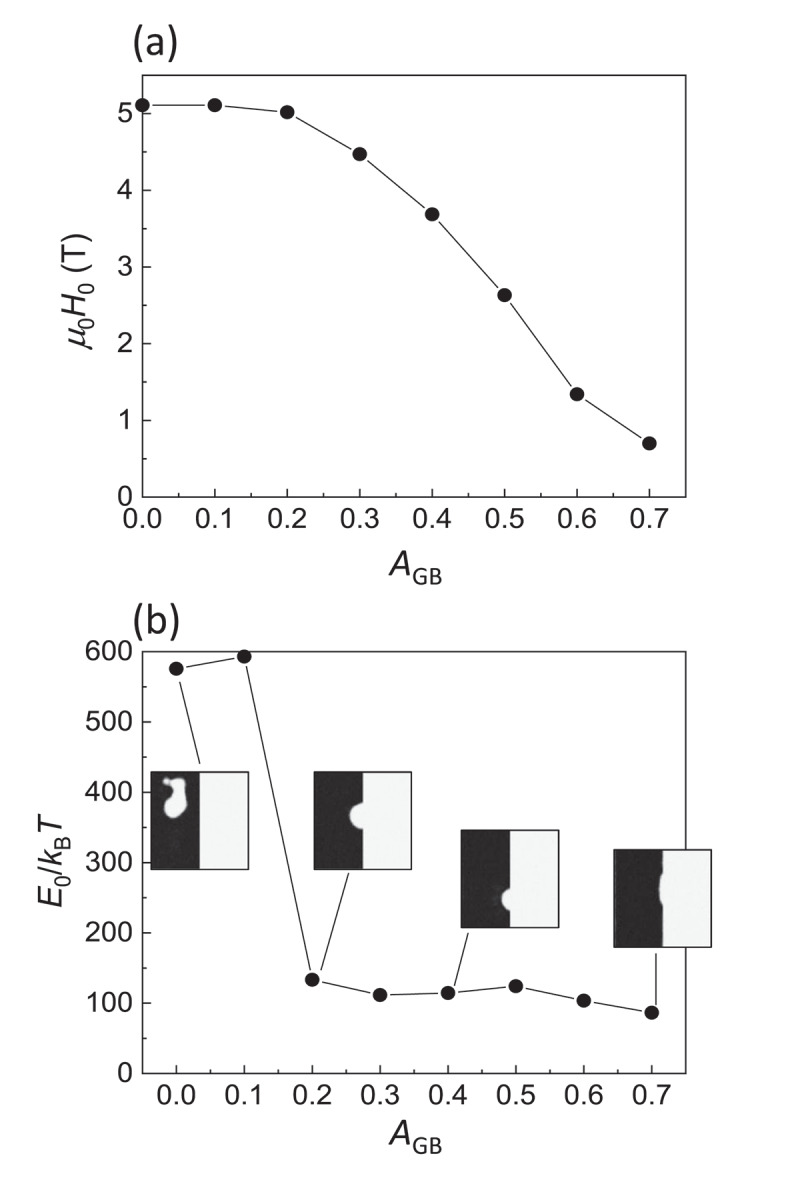
(a) *m*_0_*H*_0_ and (b) *E*_0_/*k*_B_*T* evaluated from the fitting of eq. (13) as a function of *A*_GB_. Insets in (b) are the snapshot images at which domain wall depinning just began

**Figure 10.**
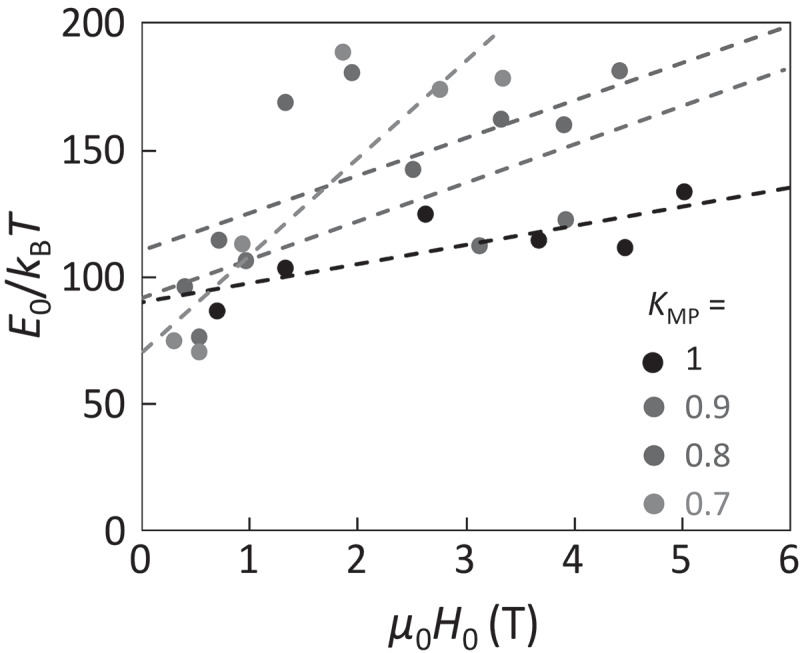
Relationship between *E*_0_/*k*_B_*T* and *m*_0_*H*_0_ obtained from the LLG simulation for various values of *K*_MP_. Broken lines are the linear fitting as eye guides [26]

To understand these behaviors, computer simulation based on the Landau-Lifshitz-Gilbert (LLG) equation was performed using MuMax3 software [48]. A two-grain model was employed, as schematically shown in Figure 8(a). The model size is 256 nm in width and 20 nm in height with 1 nm cubic meshes. The center (*xz*) plane corresponds to the grain boundary, and the right and left grains are initially set to be the up and down magnetization states, respectively. To eliminate the effect of the very strong demagnetization field from the outer boundary, periodic boundary conditions are given for the *x-, y*-, and *z*-axes. The magnetic easy axis is along the *z*-axis. A random field corresponding to a thermal energy of 300 K is given to simulate the thermally activated magnetization reversal. The magnetic anisotropy and exchange stiffness are identical to those of Nd_2_Fe_14_B [49,50]. The domain wall depinning process strongly depends on the many parameters of grain boundary phase such as magnetization, magnetic anisotropy, exchange stiffness, and thickness. In this study, for simplicity, the parameter *A*_GB_ is introduced as the dimensionless exchange stiffness at the grain boundary with respect to that of Nd_2_Fe_14_B, which involves the effects of all these grain boundary parameters. Figure 8(b) shows snapshot images of the magnetization reversal, indicating that the thermally activated domain wall depinning is initiated by the formation of a very small reversed embryo with the size of nanometer range. As well as the experiment shown in Figure 6(c), the reversal probability *P*(*H*), an example shown in Figure 8(c), is obtained as a function of *H* by 40 times repeated calculations. Thus, the values of *E*_0_ and *H*_0_ are obtained from the simulation. Figure 9 shows the thus evaluated *E*_0_ and *H*_0_ as a function of *A*_GB_ varying from 0 to 0.7. For *A*_GB_ > 0.7, the domain wall cannot be pinned at the grain boundary. *H*_0_ gradually increases with decreasing *A*_GB_ and is saturated for *A*_GB_ ≤ 0.2. On the other hand, *E*_0_ keeps almost constant for *A*_GB_ ≥ 0.2 and then rapidly increases for *A*_GB_ ≤ 0.1. The snapshot images at which the magnetization reversal just begins are shown as the insets of Figure 9(b). For *A*_GB_ ≥ 0.2, the thermally activated domain wall depinning is clearly confirmed. In contrast, for *A*_GB_ ≤ 0.1, the nucleation inside the domain occurs instead of domain wall depinning, indicating that the magnetization reversal process discretely changes from domain wall depinning to nucleation. Figure 10 shows the relationship between *E*_0_ and *H*_0_ evaluated from the simulation. In this figure, the data for *A*_GB_ ≤ 0.1 are excluded because of the different magnetization reversal process from that for *A*_GB_ > 0.2. To discuss the effect of the magnetic properties of the main phase, the magnetic anisotropy *K*_MP_ with respect to that of Nd_2_Fe_14_B is varied. For *K*_MP_ = 1 (i.e., no deterioration of magnetic anisotropy), *E*_0_ exhibits little dependence on *H*_0_. This behavior is consistent with the experimental results of Samples A and C in Figure 7. On the other hand, the slope of *E*_0_ against *H*_0_ becomes steeper with decreasing *K*_MP_. This seems consistent with that of Sample B, which exhibits the lowest *H*_c_ among the three samples studied in this work.

These results lead to some insights into thermally activated domain wall depinning. First, *A*_GB_ only affects the critical field of domain wall depinning. Second, *E*_0_ is determined by the main phase magnetic properties because *E*_0_ is the critical energy for the expansion of the domain wall depinning nucleus, which is grown inside the main phase grain. In particular, because the domain wall depinning nucleus is on the order of the domain wall thickness, *E*_0_ strongly reflects the magnetic properties of the surface of the main phase grain.

**Figure 11.**
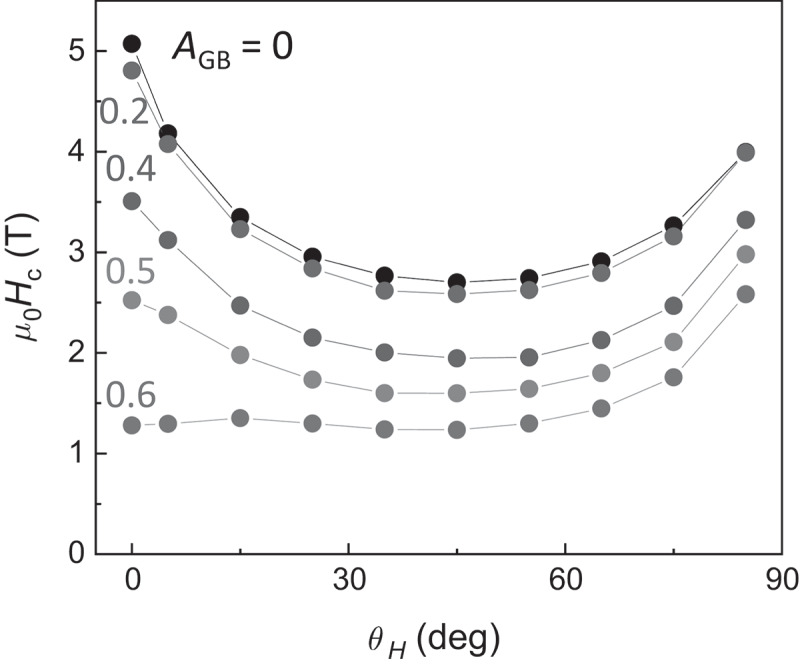
Angular dependent μ0Hc obtained from the LLG simulation for various values of *A*_GB_

Finally, the angular dependence of *H*_c_ is calculated using the two grain model. Figure 11 shows *H*_c_ as a function of the field direction *θ*_H_ with respect to the magnetic easy axis for various *A*_GB_ including the range of nucleation and domain wall depinning as discussed above. For *A*_GB_ = 0, at which the nucleation occurs inside the main phase grain, the asteroid curve-like behavior is clearly obtained. *H*_c_ at *θ*_H_ = 0 ° gradually decreases with increasing *A*_GB_, and then the angular dependence of *H*_c_ becomes close to the behavior of 1/cos*θ*_H_. As mentioned above, the magnetization reversal process changes from nucleation to domain wall depinning at *A*_GB_ = 0.2 discontinuously, and *E*_0_ suddenly changes at *A*_GB_ = 0.2, as shown in Figure 9(b). However, the angular dependence of *H*_c_ for *A*_GB_ = 0.2 still exhibits asteroid curve-like behavior. The angular dependences of *H*_c_ for *A*_GB_ = 0.4 and 0.5 are close to the asteroid curve-like behavior rather than the 1/cos*θ*_H_-like behavior. These results suggest that the identification of the magnetization reversal process of the nucleation or domain wall depinning process is quite difficult only from the angular dependence of *H*_c_.

## 4 Summary

In this paper, we reviewed the coercivity mechanism and its analyses for permanent magnets. Previously, it has been widely believed that the thermal activation process is not important in the magnetization reversal of permanent magnets because permanent magnets are bulk materials. As discussed in this paper, however, the thermal activation process, which forms a small reversed embryo with a size on the order of nanometers, plays a critical role in the magnetization reversal of permanent magnets.

We studied the macroscopic and microscopic approaches for the thermally activated magnetization reversal process in advanced Nd-Fe-B hot-deformed magnets. Through these studies, the physical picture of the thermal activation process becomes much clearer. Moreover, we would like to emphasize that the energy barrier parameters discussed in this paper strongly reflect the magnetic properties of the grain boundary phase and grain surface. In fact, the modern advanced magnets have been developed for the purpose of improving the magnetic properties of the grain boundary phase and grain surface. However, their direct evaluation is not easy. The energy barrier analysis is expected to be an evaluation method for them.
